# Body Extension by Using Two Mobile Manipulators

**DOI:** 10.34133/cbsystems.0014

**Published:** 2023-03-17

**Authors:** Yusuke Hirao, Weiwei Wan, Dimitrios Kanoulas, Kensuke Harada

**Affiliations:** ^1^ Graduate School of Engineering Science, Osaka University, Osaka, Japan.; ^2^ Department of Computer Science, University College London, London, UK.

## Abstract

This paper presents a remotely operated robotic system that includes two mobile manipulators to extend the functional capabilities of a human body. Compared with previous tele-operation or robotic body extension systems, using two mobile manipulators helps with enlarging the workspace and allowing manipulation of large or long objects. The system comprises a joystick for controlling the mobile base and robotic gripper, and a motion capture system for controlling the arm poses. They together enable tele-operated dual-arm and large-space manipulation. In the experiments, a human tele-operator controls the two mobile robots to perform tasks such as handover, long object manipulation, and cooperative manipulation. The results demonstrated the effectiveness of the proposed system, resulting in extending the human body to a large space while keeping the benefits of having two limbs.

## Introduction

Robot remote control (i.e., tele-operation) has been a popular topic in the last decades due to the massive development of speedy wireless communication and the debut of low-cost collaborative manipulators. Such remote control methods targeted at tasks that were difficult or dangerous to be directly performed by humans, or hard to be done completely autonomously. For example, several robots were developed to remotely collect information or perform manipulation tasks at the Fukushima nuclear power plant after radioactive contamination was detected [1–3]. There were also many field robots developed for collecting data at distant damaged sites caused by earthquakes or other man-made and natural disasters [4,5], medical sites without expert doctors [6,7], and outer space [8,9], to name some. The research interest on remote control has been further signified in the past 2 years as the coronavirus disease 2019 (COVID-19) pandemic restricted people’s movement. A series of robots were developed and remotely deployed at the Wuhan hospitals to assist doctors and patients [10]. Under this background, in this work, we developed a remote control system for extending human reachability and function range via two mobile manipulators. The proposed system allows to extend human body functionality to larger spaces while keeping the benefits of having two limbs [11].

The previously developed remote robot control systems tend to have a mobile base and one or two robotic arms mounted on the base. They also usually require a special-purpose tele-operation interface [12–14] to map human tele-operator motion to the robot. Those systems had limited work space. The tele-operation interface (leader) and remote robot (follower) pair was bulky and not easy to be decoupled and redeployed. Different from the previously developed systems, we propose the use of Inertia Measurement Unit (IMU)-based motion capture gloves and joysticks to remote control two mobile manipulators. The gloves and joysticks are decoupled from the mobile manipulators. They are generic, commercially available, and appropriate for remote controlling different robots. The two mobile manipulators have independently movable bases. They can be separated far from each other; thus, they substantially enlarge the work space and extend the reachability of a human tele-operator. In detail, we use the motion capture system to track the human tele-operator’s two hand poses and use the obtained poses to control the poses of respective robot arms. The two joysticks are used to control the two mobile bases.

Separating the two arms into independently movable bases for motion-tracking-based teleoperation has the following inherent issues: (a) The robots’ base coordinate systems may have very different poses and are difficult to be mapped to a human tele-operator’s two arms. (b) The robot may move dangerously when the human tele-operator performs exceptional motions during tracking. (c) Remote surveillance could be challenging due to the large operation ranges. We developed special routines to solve the first two problems and took advantage of the robots’ hand-mounted cameras to overcome the last challenge. We tested the proposed system by performing tasks, such as single-arm manipulation, two-arm collaborative manipulation of long objects, two-arm cooperative assembly, and two-arm handover. The results demonstrated the effectiveness of the proposed system in extending human functions.

The organization of this paper is as follows. The Related Work section presents related work. The System Architecture and Control Methods section presents the hardware architecture of the proposed system. The Inherent Issues and Solutions section presents control methods and exception handling algorithms for making the system robust. The Experiments and Analysis section demonstrates and analyzes experiments results. Conclusions are drawn in the Conclusions section.

## Related Work

In this section, we review several remotely operated robotic systems, with a special focus on those that involve motion capture systems, joysticks, and mobile manipulators.

For single-arm mobile manipulators, Annem et al. [15] presented a tele-operated mobile manipulator for tending machines and transporting parts in manufacturing applications. The mobile manipulator is controlled through a computer-based graphical interface. It accepts high-level goals from human tele-operators and generates navigation and manipulation motion, considering various sensor feedback. Su [16] used mixed reality devices to tele-operate a mobile manipulator for manufacturing tasks. The developed remote control method helped to improve the welding results of unskilled workers. Ha et al. [17] developed a semi-autonomous tele-operation system that used optical tracking-based motion capture system to obtain human motion and used multimodal equipment such as haptic devices and head-mounted display for feedback. Their remote robot is a submarine vehicle with a manipulator mounted on its front surface. The authors carefully designed the command mapping between a human body and the mobile manipulator. Bejczy and Szakaly [18] used multiview cameras to assist the tele-operation of a mobile manipulator. They practiced their system with LEGO brick assembly tasks. Mast et al. [19] developed different interfaces with different autonomy levels to remotely control a mobile manipulator to assist elderly people at home. The interface was a touching pad for caregivers and a computer with haptic devices for tele-assistance of professionals.

For dual-arm mobile manipulators, Malysz and Sirouspour [20] presented the asymmetric semi-autonomous tele-operation framework and practiced it for systems with unbalanced leader and follower correspondences. They especially examined the effectiveness by using a dual operator to remotely control a dual-arm mobile robot. Song et al. [21] developed a shared-control method [22] for tele-operating a dual-arm mobile robot. The controller automatically switches between robot autonomy and human skills, considering weights and priority. Garcia et al. [23] improved a shared-control method by applying remote center of motion constraints to a tele-operated robot arm. Bennet et al. [24] used an exo-suit to tele-operate a dual-arm mobile robot. They studied the interaction patterns of a human and the tele-operated robot, and compared them with a fully autonomous alternative. Lv et al. [25] used an IMU-based motion capture system to tele-operate a dual-arm collaborative robot for dementia care in home environments. Bandala et al. [26] developed a graphical user interface with two-dimensional vision data for tele-operating a dual-arm hydraulic robot. They could perform heavy tasks such as pipe cutting and moving large objects in nuclear environments. Buss et al. [27] used two dual-arm mobile robots independently controlled by two human operators to perform collaborative tasks in a remote environment. The robots could manipulate large and long objects because of the independent mobility. The human operators interacted with each other by visual and auditory feedback. The remote control policy was improved in [28] for DLO-in-hole tasks (deformable linear object) while considering the shared-control paradigm. Zhou et al. [29] used virtual reality devices to control a dual arm for pipe operation tasks.

The following studies focus on interfaces and considered a combination of autonomy and tele-operation for dual-arm control. Mortimer et al. [30] studied the relationship between robotic characteristics and the necessary interface configurations required for tele-operation. A toolbox was developed to recommend the configurations, which was later integrated as a part of a dynamic virtual reality user interface for tele-operating heterogeneous robot teams [31]. Wang et al. [32] compared the effectiveness of joint space mapping and task space mapping in tele-operating a manipulator and concluded that task space mapping is more friendly to human tele-operators. Wildenbeest et al. [33] studied the influence of haptic feedback quality on the performance of tele-operated assembly. The authors concluded that low-frequency haptic feedback was the primary factor for good tele-operation performance. Further improving feedback quality had marginal contribution. Talasaz et al. [34] developed a visualization system for manipulation forces and compared direct and visual force feedback in a tele-opearated dual-arm suturing task. Sun et al. [35] used a single leader to tele-operate two robot manipulator followers. The cooperatively manipulated object was considered as the control target while respective manipulators moved autonomously. Zhou et al. [36] developed a dual-arm tele-operation system by using exactly the same architecture on the leader and follower sides. Liu et al. [37] used electroencephalography (EEG) signals to tele-operate a dual-arm robot for pick and place tasks. Similar studies included [38,39], which further improved EEG-based tele-operation with learning or feedback. Tam et al. [40] presented a review of the various methods for extracting data from neural signals like the EEG. Nicolis et al. [41] used one arm of a dual-arm robot to perform automatic visual servoing while tele-operating the other arm. The automatic arm helped provide occlusion-free visual feedback to human tele-operators. Bai et al. [42] combined motion planning and tele-operation for dual-arm twisting. One of the robot arms was controlled by autonomous optimized motion planning. The other arm was telecontrolled by a human.

Compared with the above studies, our main difference is the use of two mobile manipulators to extend the reachability and functionality of a human body. We used an IMU-based motion capture device to capture a human’s two hand poses and thus control the two mobile manipulators in the task space. We also used two joysticks for controlling the two mobile bases and robotic end-effectors (grippers). The two mobile manipulators are assumed to extend the human operator’s workspace range. The human operator observes the two manipulators directly to understand the working states. We developed detection algorithms to watch and ignore the exceptional motion from a human tele-operator. The detection algorithms not only helped make the remote control robust but also permitted repeatedly disconnecting and recapturing the robot arms. A human tele-operator could take advantage of the disconnection and recapture to recalibrate the discrepant coordinates between the two arms. We also used hand-mounted cameras and independent mobile robot arms to display grasping details to remote humans. It helped overwatch the operation performance in large work ranges. These detailed solutions are inherent to the independent mobile bases and motion tracking-based interface. They were less mentioned in previous studies and from the main contributions of our work.

## System Architecture and Control Methods

**Fig. 1.**
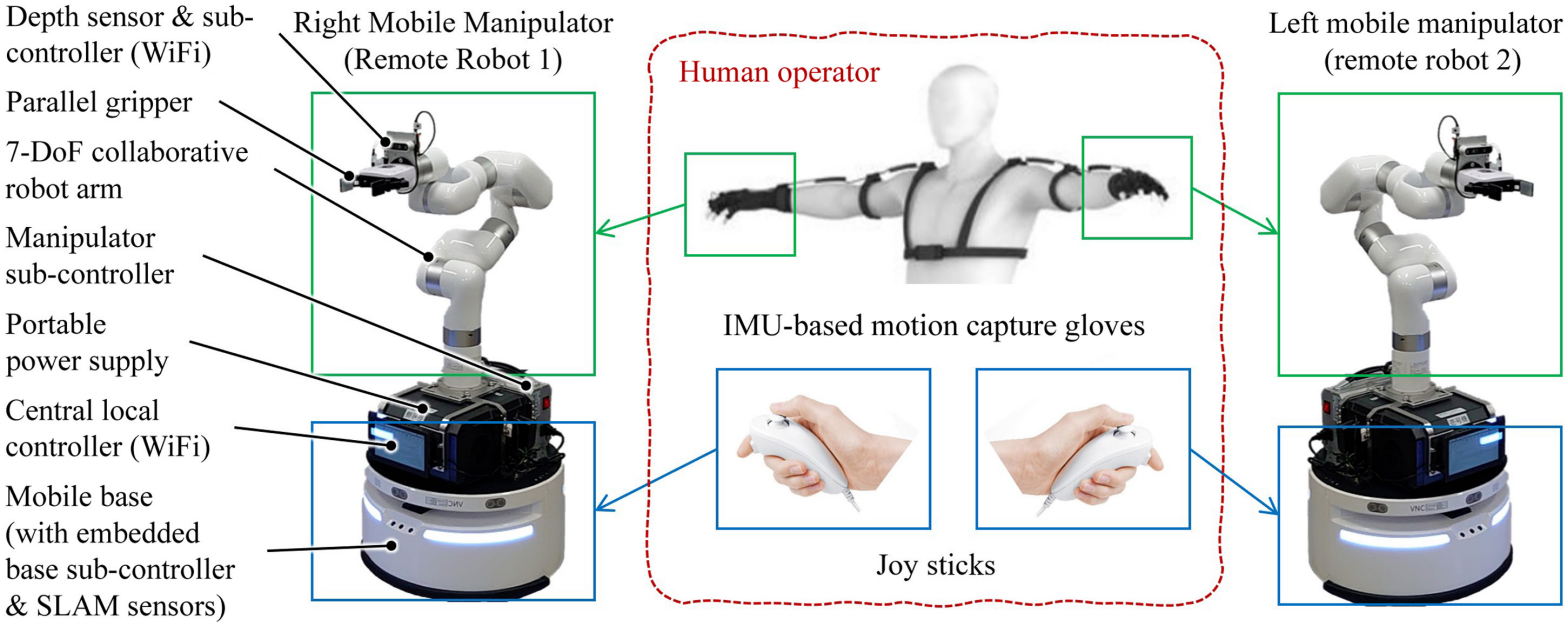
Components of the developed body extension system.

Figure 1 shows the various components of the developed system. The central red dashed box shows the tele-operation interface on the human tele-operator side. It includes two IMU-based motion capture gloves (IMU-based motion capture: Perception Neuron, Noitom Ltd.) and two joysticks (joysticks: Nintendo Wii Nunchunk). The IMU-based motion capture gloves help measure the position and orientation of the human tele-operator’s two hands. Each joystick comprises one joystick button and two pressing buttons. They are used for controlling the base movement and gripper actions, respectively.

The two remote mobile manipulators are illustrated on the right and left sides of the human tele-operator in the figure. Each mobile manipulator comprises a non-holonomic mobile base and a 7-DoF (degree of freedom) collaborative robot arm. Both of these are independent commercial products on the market (robot arm: UFACTORY xArm 7; mobile base: YUNJI Water 2). The manipulator and the mobile base are connected to each other by a steel frame. A portable power supply is fixed inside the frame to provide power supply to the robot arm (portable power supply: EENOUR EB120). A central local controller is mounted at the front side of the portable power supply to receive and transfer commands to the manipulator and mobile base through WiFi. It is inside the display shown in the figure. The display helps monitoring the central local controller’s status. The manipulator and mobile base have their subcontrollers. They are mounted at the back side of the portable power supply and embedded inside the mobile base. The central local controller plays the role of a higher-level node for communicating with and coordinating the two respective controllers. Each mobile manipulator has a gripper as its end-effector. A depth sensor with a subcontroller computer is installed between a gripper and an arm for visual sensing. The depth vision system also receives commands and distributes data through WiFi.

**Fig. 2.**
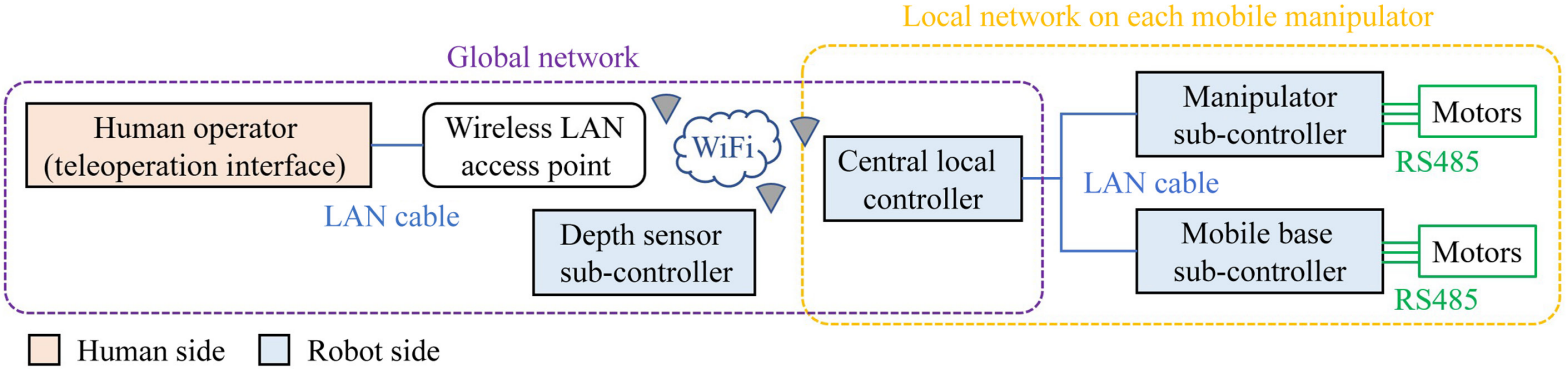
Network architecture of the developed body extension system.

Figure 2 shows the network architecture of the developed system. The human interface has a computer that is connected to a wireless local area network (LAN) access point by a LAN cable. The central local controller and the depth sensor subcontroller are connected to the wireless LAN access point by WiFi. The manipulator subcontroller and the mobile base subcontroller are connected to the central local controller by LAN cables. They connect to the motors of the manipulator and mobile base by RS485. There are three networks across the system. The first one is the global network connecting the human side and robot side. The other two are the networks on each of the two mobile manipulators. The wireless LAN can be replaced by long-range communication modules like LTE for field tasks.

**Fig. 3.**
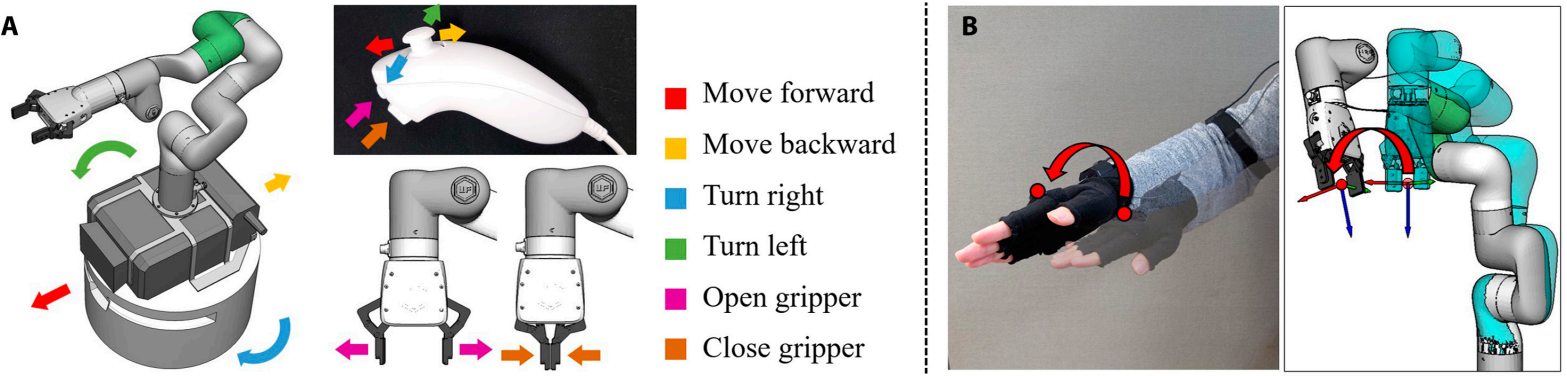
Motion mapping between the interface and the robots. (A) The joystick button and two pressing buttons of a joystick controller are mapped to the motion of the mobile base and action of the gripping end-effector, respectively. (B) The pose of the IMU sensor is mapped to the pose of the robot gripper.

The motion mapping between the interface and the robots is illustrated in Fig. 3. For the mobile base, we map the joystick button and two pressing buttons of a joystick controller to the motion of the mobile base and action of the gripping end-effector, respectively. For the robot arm, we map the pose of the IMU sensor on the motion capture glove to the pose of the robot gripper. We track the pose of the human hand and use an improved LM (Levenberg-Marquardt) method [43,44] to continuously solve the robot inverse kinematics following the tracked human hand pose. The following equations show the details of the improved LM method:

$$\Delta q=J^{\#}e+\left(I-J^{\#}J\right)C,$$ (1)

$$\text{where}\;J^{\#}=W\;J^{T}{\left(\mathrm{JW}\;J^{T}+\lambda^{2}I\right)}^{-1}$$ (2)

$$\;C=-\left(I-H_{w}\right)\left(2*q-q_{\text{mdl}}\right)/q_{\text{rng}}$$ (3)

where ***e*** is the position and rotation difference between two adjacent human hand poses. ***J*** is the Jacobian matrix. ***J***^#^ is the weighted pseudo-inverse considering a damper item *λ*^2^***I*** to avoid singularity. ***W*** is the weight matrix. (***I*** − ***J***^#^ ***J***)***C*** is the null space constraint. It is used to make sure that the joint angles do not run out of the effective ranges represented by ***q****_rng_*. ***q****_mdl_* is the middle value of each joint range. ***I*** − ***H****_w_* is the activation matrix for clamping velocity. ***C*** is the clamping matrix for cutting off extraneous joint movement. The details of the clamping matrix can be found in [45]. It helps improve the original LM method by watching the joint angle deviations from their median values. A significant deviation toward the joint range boundaries will lead to a significant increase in the null space projection and, thus, a high gain to avoid out-of-range failures. Δ***q*** is the differential joint angles for updating the robot configuration and following the human motion.

**Fig. 4.**
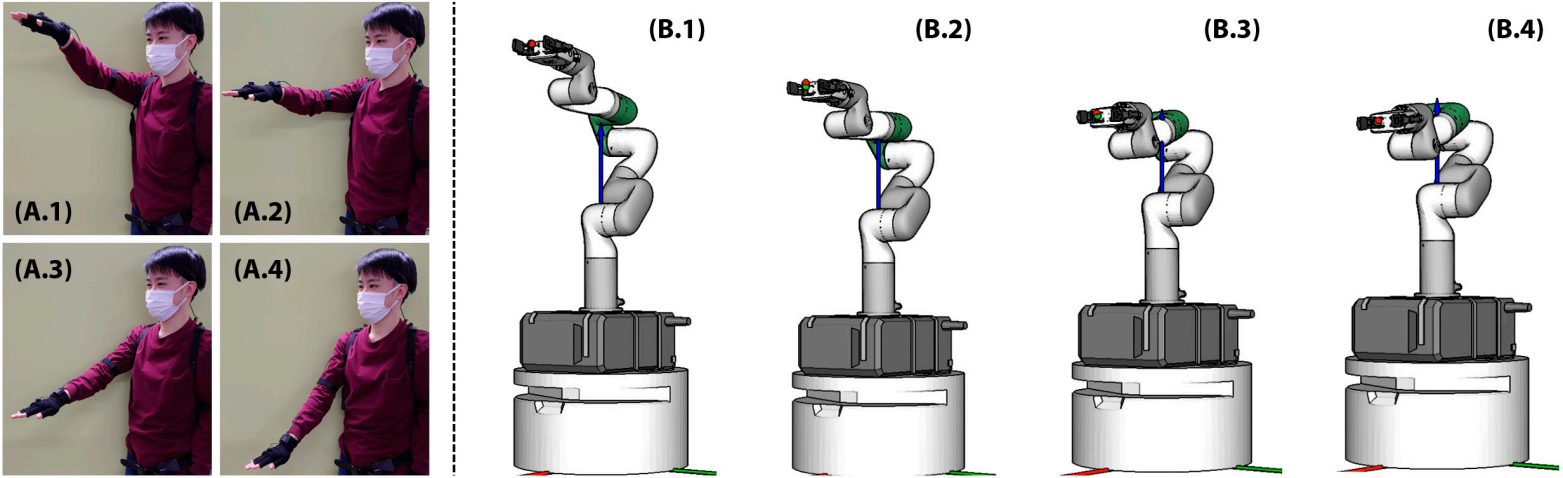
(A and B) Motion mapping results using the improved LM method.

The equation is repeatedly applied to each tracked hand pose frame to obtain a continuous manipulator movement. Figure 4 shows the results. Note that we could also take into account environmental obstacles and develop a semiautomatic tele-operation system that can smartly avoid collisions. Particularly, we may either implement the collision avoidance by including distances to obstacles in the ***C*** item of (***I*** − ***J***^#^ ***J***)***C*** or add a second subtask in the null space of the current joint range task. However, the current tele-operation interface does not provide a port for signaling obstacle distances or obstacle avoidance commands to guide the redundant joints. It will be an open problem for further studies.

## Inherent Issues and Solutions

Using the proposed interfaces, tele-operating two independent mobile manipulators has inherent issues. This section discusses these issues and presents our solutions.

### Exceptional tele-operation motion

**Fig. 5.**
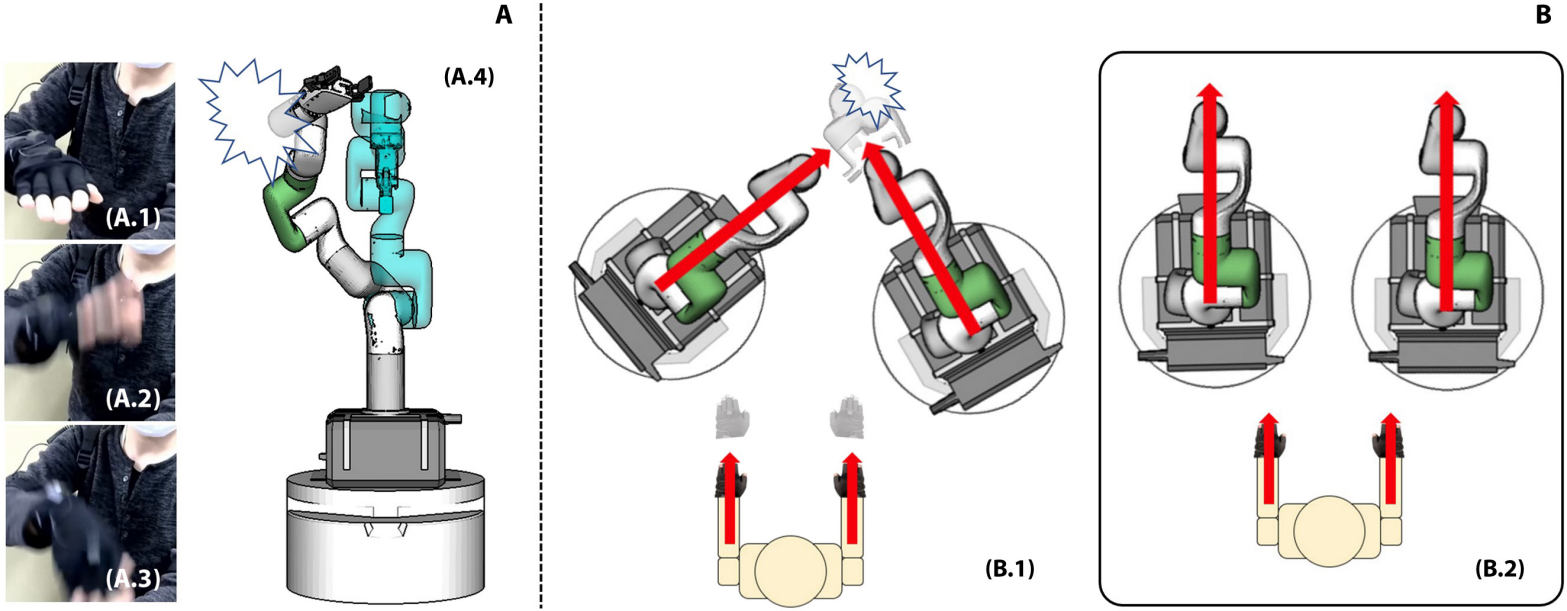
(A and B) Motion mapping results using the improved LM method.

The first issue is the exception motion of a human tele-operator. Since we track the human hand motion continuously and map the continuous hand pose changes to the robot, the robot may move dangerously when the human tele-operator performs exceptional tele-operation motions. Figure 5A shows an example of such exceptional motion. The human tele-operator randomly shook their hands during tele-operation (Fig. 5A.1 to A.3), and the robot moved significantly and crashed itself (Fig. 5A.4).

In detail, we keep watching the expected manipulator joint angle changes to avoid exceptional human motion. If the expected manipulator joint angle changes are larger than some given threshold values, the system determines that the tele-operator is performing an exceptional motion and stops following the tracked trajectories. After disconnecting the robot and the tele-operator, the system continues to watch if the tele-operator recatches the robot. It examines the position error and rotation error between the coordinate of the manipulator end-effector when the tele-operation was disconnected and the coordinate of the tele-operator’s hand. If the errors are less than some given thresholds, the system resumes connecting the tele-operator and the robots, and drives the robots following the tracked tele-operator trajectories.

### Calibration before dual-arm collaboration

The second issue is the discrepancy between the human tele-operator and the two robotic manipulators. Figure 5B.1 shows an example. The motion of the human tele-operator’s two hands is mapped to the robot manipulators considering the relative changes between every two adjacent tracking frames. If the initial poses of the two mobile manipulators are not well calibrated with the initial human arm poses, the coordinated motion of the two human hands might be mapped to a wrong coordinated motion on the robot side. A representative failure is shown in Fig. 5B.1, where the two robot manipulators run into collision, although the human tele-operator moved their two hands forward in parallel. Other adverse effects include strange tele-operation feelings caused by the inconsistent facing directions and cramped tele-operation workspace. For this reason, the human tele-operator must carefully adjust the two mobile robots and formulate them into a well-coordinated pose before performing collaborative tasks between the two arms. Figure 5B.2 shows an example of a formulated and well-coordinated robot and human configuration.

Despite the illustration in Fig. 5B.2, we do not have an automatic routine or a specific human pose for carrying out the calibration in our implementation. The human tele-operator may find his or her most comfortable pose for calibration and is allowed to calibrate repeatedly during tele-operation to successfully finish the goal tasks. The repeated calibration is essentially carried out as a callback to the exception routine. The human tele-operator may actively perform exception motion to disconnect from the robot, switch (calibrate) to a comfortable pose, and recatch the robot for continued teleoperation tasks.

It is worth nothing that a bad initial calibration will lead to awkward human poses and, thus, cramped tele-operations. It will not cause failures since the human tele-operator may recalibrate (reset and recapture) the robot whenever necessary. However, he or she may bother to carry out recalibration multiple times to finish a task.

## Experiments and Analysis

In this section, we carried out experiments with different levels of difficulties to examine the effectiveness of the developed system.

First, we examine the precision of our improved LM method. Table shows the position error with and without the improved LM method. Here, “without the improved LM method” means solving each Inverse Kinematics IK independently using the solver provided by the robot. Compared with the iterative LM solver, the independent method has a much larger error norm. The proposed iterative method is thus considered a better choice for continuous tele-operation.

**Table.** Errors of the methods with and without the iterative LM solver.

	With LM				Without LM			
	*x*	*y*	*z*	Norm	*x*	*y*	*z*	Norm
Error [m]	−1.35231e−5	8.357e−4	6.719e−4	0.00107	0.0464	−0.0274	−0.0734	0.0911

**Fig. 6.**
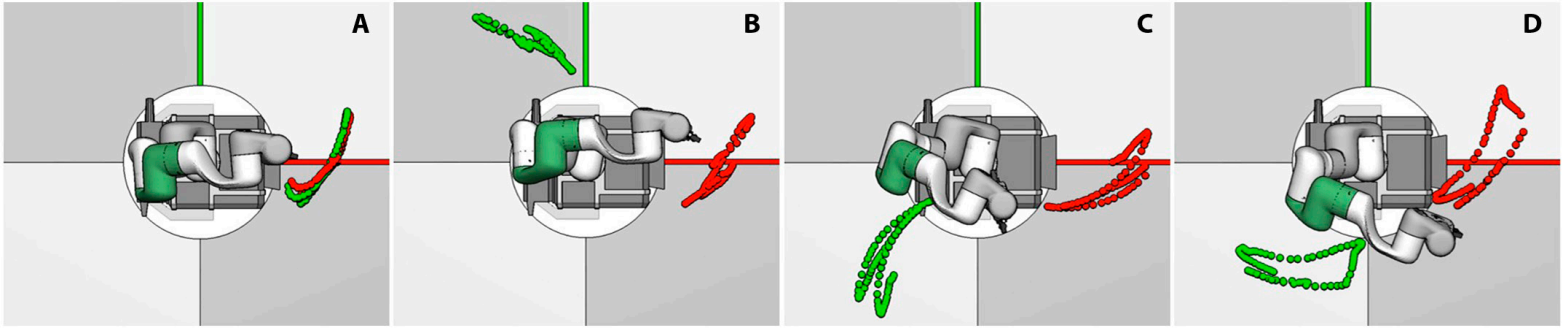
Comparison between the human hand trajectories (green) and the tele-operated robot trajectories (red). (A to D) Results when the human tele-operators performed tele-operation while facing at different forward directions.

Second, we study the motion mapping performance by visualizing the human hand trajectories and the tele-operated robot trajectories. Figure 6 shows the visualization results. The green trajectories are from the human hands. The red trajectories are from the robot manipulators. The four subfigures (Fig. 6A to D) show the results when the human tele-operators performed tele-operation while facing at different forward directions. Since the improved LM method is carried out iteratively considering the local changes between adjacently tracked poses, the robot motion is irrelevant to the human tele-operator’s facing direction. The robot manipulator always performed trajectories similar to the human by starting from its initial pose.

Third, we asked a human tele-operator to use the system to perform three manipulation tasks. In the first task, the human tele-operator was asked to operate one of the manipulator to pick up a coffee bottle. In the second task, the human tele-operator was asked to operate two mobile manipulators to perform a handover task. In the third task, the human tele-operator controlled the two robots to pick and place two sticks with different lengths and examined the system’s ability to adapt to varying scenario scales. All the tasks were conducted with the tele-operator and robot facing each other. All the tasks could be successfully performed in the experiments, although the tele-operator needed several corrections before reaching a satisfying pick-up or handover state. For example, the second task was unsuccessful initially and had to be corrected several times to avoid collisions between the manipulators during the handover. Also, the tele-operator may need much time to move the robot arms carefully and ensure good coordination between an arm and an object, or between two arms. The time required for the three tasks was around 50 s, 120 s, and 50 s (long stick)/70 s (short stick), respectively. The time costs were higher when both arms were involved. Note that the tele-operator did not practice the usage before the experiments. As tele-operator performs more trials, he or she should be accustomed to the operation, and the time costs would be decreased.

**Fig. 7.**
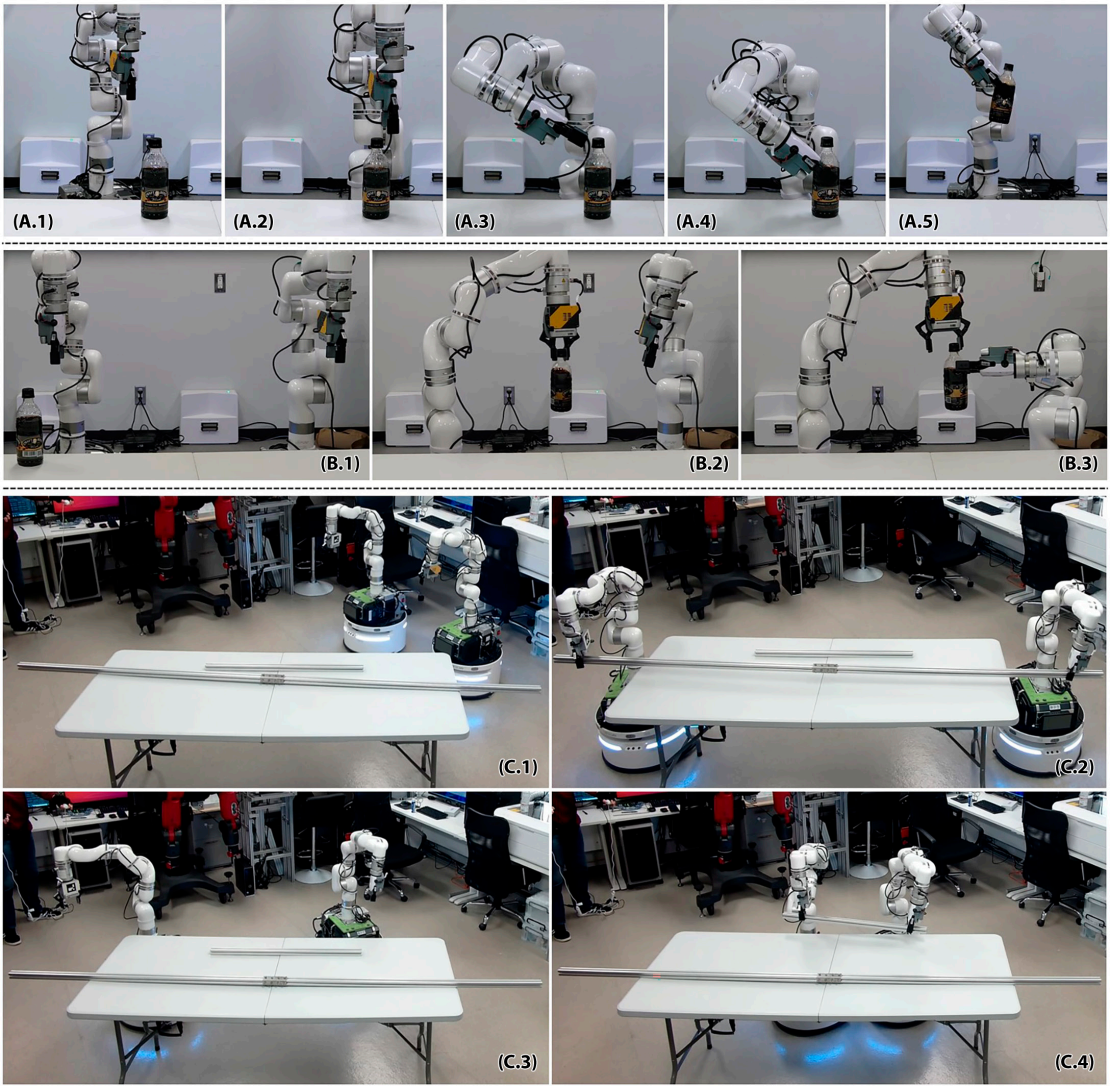
Snapshots of tele-operating the robots to perform three manipulation tasks. (A) Pick up a coffee bottle. (B) Hand over a coffee bottle. (C) Pick and place long and short sticks.

Some snapshots are shown in Fig. 7. The complete results can be found in the supplementary video. Especially for the latter three tasks, the human tele-operator needs to carefully calibrate the two arms before performing the tele-operated handover, as discussed in the Calibration before dual-arm collaboration section. Otherwise, it would be difficult for the two hands to precisely cooperate with each other. The handover task had a higher requirement for calibration compared to the collaborative stick transportation. The longer stick in the third task was less costly because the coordination errors had less influence on each individual robot when they were far from each other, and the human saved time from calibration.

## Conclusions

In this paper, we presented a tele-operation system for extending the body of a human tele-operator. The system comprised two mobile manipulators that can move independently in a large workspace. The human tele-operator could thus take advantages of the independence to manipulate objects in a long range. Also, since there are two manipulators, the presented system could keep the advantages of having two arms while extending the human body functions. Different levels of experiments were performed to examine the effectiveness of the proposed system. The results confirmed our expectations.

There remain several open problems for future development. First, the two independent mobile bases lead to a mismatch between the direction of the human tele-operator’s arms and the manipulators. We currently rely on the human tele-operator’s repeated calibration to solve the problem. A method with less calibration burden is highly demanded. Second, the joystick controller must be held horizontally in a human hand due to the need for tracking the IMU sensors on the hand back. The operation was different from the usual way of holding the joystick. Using a joystick with an embedded IMU sensor may help solve the problem. Third, we can only implement pick-and-place motion with compliant hand poses due to the coordination errors between the two arms. It is advisable to have a high-level synchronized control mode where the tele-operator controls the object poses, and the two robots generate synchronized coordinated motion automatically. Fourth, the presented system does not have a feedback interface for tele-operation in a distant and unseen workspace. Future work will also include developing additional components for such scenarios.
